# Interaction of Diamine Oxidase with Psychostimulant Drugs for ADHD Management

**DOI:** 10.3390/jcm12144666

**Published:** 2023-07-13

**Authors:** Yaiza Tobajas, Marc Alemany-Fornés, Iris Samarra, Jordi Romero-Giménez, Maria Tintoré, Antoni del Pino, Núria Canela, Josep M. del Bas, Nàdia Ortega-Olivé, Carlos de Lecea, Xavier Escoté

**Affiliations:** 1 Eurecat, Centre Tecnològic de Catalunya, Nutrition and Health, 43204 Reus, Spain; yaiza.tobajas@eurecat.org (Y.T.); jordi.romerog@eurecat.org (J.R.-G.); nadia.ortega@eurecat.org (N.O.-O.); 2 DR Healthcare-AB Biotek, 08017 Barcelona, Spain; malemany@dr-healthcare.com (M.A.-F.); maria.tintore@abbiotekhealth.com (M.T.); carlos.delecea@abbiotekhealth.com (C.d.L.); 3 Centre for Omic Sciences (COS), Joint Unit URV-EURECAT, Unique Scientific and Technical Infrastructures (ICTS), Eurecat, Centre Tecnològic de Catalunya, 43204 Reus, Spain; iris.samarra@eurecat.org (I.S.); antoni.delpino@eurecat.org (A.d.P.); nuria.canela@eurecat.org (N.C.); 4 Eurecat, Centre Tecnològic de Catalunya, Biotechnology Area, 43204 Reus, Spain; josep.delbas@eurecat.org

**Keywords:** ADHD, DAO, histamine intolerance, psychostimulant drugs

## Abstract

Histamine intolerance occurs when there is an imbalance between histamine production and the capacity for histamine degradation. Diamine oxidase (DAO) is the main enzyme for the catabolism of ingested histamine degradation in the gastrointestinal tract and its deficiency has been linked to allergy-like symptoms. Psychostimulant drugs are commonly used to treat Attention Deficit Hyperactivity Disorder (ADHD), but their interaction with DAO is not well characterized. In this work, we evaluated the effects of psychostimulant drugs (methylphenidate and lisdexamfetamine) on in vitro DAO activity and in the human cell line of enterocytes (Caco-2), evaluating DAO expression (mRNA and protein) and DAO activity. Methylphenidate and lisdexamfetamine did not repress the in vitro DAO activity. In addition, in Caco-2 cells, lisdexamfetamine promoted a strong upregulation of DAO mRNA levels, whereas methylphenidate tended to induce DAO activity. To sum up, methylphenidate and lisdexamfetamine treatments do not reduce DAO activity. These findings could be useful for physicians prescribing these two drugs to ADHD patients affected by DAO deficiency.

## 1. Introduction

Attention Deficit Hyperactivity Disorder (ADHD) is a neurodevelopmental disorder that typically manifests in childhood and can persist into adulthood, characterized by symptoms of inattention, hyperactivity, and impulsivity that can interfere with a person’s daily functioning and relationships [1]. The symptoms can vary in severity and can have a significant impact on a person’s academic, social, and occupational success [2]. The current clinical protocols for the diagnosis and treatment of ADHD involve medication with psychostimulants, such as methylphenidate and lisdexamfetamine and behavioral therapy [3]. It is important to note that there is no cure for ADHD [4], but with proper management, individuals with the condition can learn to manage their symptoms and lead successful, fulfilling lives.

Histamine is a neurotransmitter known to be an important mediator of many biological processes: gastric acid secretion, inflammation, neuromodulation and regulation of immune function [5]. This amine is produced by the body itself and ingested from food. However, when free histamine concentrations are high, they trigger a series of unwanted side effects known as histamine intolerance (HIT) that can cause a variety of symptoms such as headaches, skin rashes, and gastrointestinal distress [6,7]. The main route of breaking down histamine in the gut is through the diamine oxidase (DAO) enzyme and DAO deficiency is a common cause of HIT [8]. HIT and DAO deficiency have been linked to a variety of conditions, including irritable bowel syndrome (IBS), migraines, and allergic reactions [9]. While there is no cure for HIT or DAO deficiency, dietary modifications and supplements such as DAO enzymes can help manage symptoms and improve quality of life for those patients [10]. In addition, HIT and ADHD share some common traits, including allergy-like symptoms and gastrointestinal (GI) symptoms [11]. Research has found that individuals with ADHD often experience allergy-like symptoms, such as nasal congestion, runny nose, and sneezing, as well as GI symptoms such as abdominal pain, bloating, and diarrhea [11]. These symptoms are similar to those experienced by individuals with HIT. One possible explanation for the overlap in symptoms between HIT and ADHD is the role of histamine in the body. In addition, there is some evidence to suggest that histamine levels may play a role in ADHD [12,13,14], although the nature of this relationship is not yet well understood. There is limited research on the prevalence of DAO deficiency specifically in individuals with ADHD. However, some studies suggest that DAO deficiency may play a role in the development or exacerbation of ADHD symptoms [15,16,17,18]. In this sense, due to the high prevalence of ADHD and the relationship that is being observed with the DAO deficiency, this connection has focused the attention on the field. Thus, Professor Blasco-Fontecilla, presented at the National Congress of the Spanish Association of Child and Adolescent Psychiatry that 77% of ADHD patients present genetic variants in the AOC1 gene associated with the reduction of the activity of the DAO enzyme, and that 15.9% presented variants associated with a severe reduction [19]. It should be noted that this situation may be accentuated because some drugs for the treatment of ADHD can interfere with DAO activity. However, recent research suggests that eating patterns and dietary interventions may be a complementary strategy for the management of ADHD [20], which could connect the decrease in histamine with a better progression of the disease. For this reason, more research is needed to fully understand the potential benefits of DAO supplementation for ADHD management.

In addition, some pharmaceutical drugs may interact with DAO inhibiting its activity [21], and this can affect histamine levels and contribute to adverse drug reactions such as allergic reactions or gastrointestinal symptoms. However, while there is some evidence for these interactions in vitro, the clinical significance of DAO-drug interactions is not yet well understood [22]. There is limited information available on the potential interactions between DAO and pharmaceutical drugs used in the management of ADHD. To date, there have been no studies specifically investigating the interactions between DAO and ADHD medications, and the current literature on DAO interactions with other pharmaceuticals is limited and mostly based on in vitro studies. ADHD medications, such as stimulants (e.g., methylphenidate and amphetamines), are generally thought to act primarily on neurotransmitters such as dopamine and norepinephrine [23], rather than histamine, which is the substrate of DAO. Therefore, it is unlikely that these medications would directly affect DAO activity. However, it is possible that medications used in the management of ADHD could indirectly affect histamine levels and DAO activity through other mechanisms. For example, some ADHD medications can affect appetite [24] and sleep [25], both of which can indirectly affect histamine levels. Overall, while there is currently no direct evidence of interactions between DAO and ADHD medications, it is possible that these medications could indirectly affect histamine levels and DAO activity. However, more research is needed to fully understand the potential interactions between DAO and ADHD medications, how these interactions affect histamine levels and to identify individuals who may be at increased risk for adverse drug reactions due to DAO-drug interactions.

Thus, this work evaluated for the first time the interaction effects of DAO with the most common psychostimulant drugs used to treat ADHD, methylphenidate and lisdexamfetamine. Taking into consideration the importance of ADHD treatment together the neurotransmitter histamine levels, we hypothesized that these psychostimulant drugs may modulate the DAO activity, which should be considered by the physicians when prescribing specific treatments for ADHD. To validate this hypothesis, we assessed the interaction of DAO with methylphenidate and lisdexamfetamine by in vitro DAO activity studies by direct inhibition with commercial and metabolized drugs, and by evaluation of this interaction in an intestinal epithelium human cell line of colonocytes at mRNA, protein, and activity level.

## 2. Materials and Methods

**Analysis of DAO activity inhibition by psychostimulant drugs**

Methylphenidate and lisdexamfetamine were selected as the standard drugs for treating ADHD with psychostimulant medication. These drugs were prepared at two concentrations (1 mM and 200 µM), and 100 µL of each drug were mixed in a glass bottle with 100 mL of 0.1 mg/mL DAO in a 0.05 M phosphate-buffered solution (pH 7.2) (final drug concentration was tested at two levels 1 µM and 200 nM). Samples were placed in a water bath orbital shaker for 1 h (37 °C, 40 U/min). To start the enzymatic reaction, 500 µL of 9000 µM histamine were added to the sample at a final concentration of 45 µM. All the samples were kept in constant incubation and 200 µL aliquots were progressively extracted at different sampling times (t = 0, 0.5, 1, 1.5, 2, and 3 h) [26]. To stop the enzymatic reaction, 15 μL of 2 N perchloric acid solution was added to the extracted aliquot. Internal standard (10 μL of 50 μg/mL Histamine-d4) was also added. Samples were mixed with a vortex mixer and centrifuged for 5 min at 4 °C and 15,000 rpm. The supernatant was diluted (20 μL in 980 μL of 0.1% formic acid in acetonitrile) and transferred to glass vials for LC-MS/MS analysis. Two positive control samples were performed with DAO (0.1 mg/mL) and aminoguanidine (final concentration of 20 nM) in a DAO solution (0.1 mg/mL) following the same methodology.

**Analysis of DAO activity inhibition by metabolized psychostimulant drugs**

To understand whether modifications of methylphenidate and lisdexamfetamine produced effects on DAO inhibition, they were treated with hepatic microsomes to simulate the metabolization of both compounds by the liver [27]. For this, 183 µL of 0.1 M phosphate-buffered solution (pH 7.4) were mixed with 5 µL of microsomes (20 mg/mL; Fisher Scientific, Waltham, MA, USA) and 2 µL of each compound (20 mM, for both methylphenidate and lisdexamfetamine, final concentration of 1 µM). The mixture was pre-incubated in water bath for 5 min, and then the reaction was started with the addition of 10 µL 20 mM NADPH. Samples were incubated in a water bath orbital shaker for 1 h (37 °C, 40 U/min). Then, the reaction was stopped by the addition of 200 µL acetonitrile. Samples were vortexed, centrifuged and 100 µL of the supernatant was used as the “test drug” for the enzymatic assay. A total of 100 µL of each drug were mixed in a glass bottle with 10 mL of 0.1 mg/mL DAO in a 0.05 M phosphate-buffered solution (pH 7.2). Samples were placed in a water bath orbital shaker for 1 h (37 °C, 40 U/min). To start the enzymatic reaction, 50 µL of 9000 µM histamine were added to the sample at a final concentration of 45 µM. All the samples were kept in constant incubation and 40 µL aliquots were progressively extracted at different sampling times (t = 0, 0.5, 1, 1.5, 2, and 3 h) [26].

To stop the enzymatic reaction, 3 μL of 2 N perchloric acid solution was added to the extracted aliquot. Internal standard (10 μL of 10 μg/mL Histamine-d4) was also added. Samples were mixed with a vortex mixer and centrifuged for 5 min at 4 °C and 15,000 rpm. The supernatant was diluted (20 μL in 980 μL of 0.1% formic acid in acetonitrile) and transferred to glass vials for LC-MS/MS analysis. Two positive control samples were performed with DAO (0.1 mg/mL) and aminoguanidine (final concentration of 20 nM) in a DAO solution (0.1 mg/mL) following the same methodology [26].

**LC-MS/MS methodology**

The chromatographic separation was performed with a gradient detailed in Supplementary Table S1. Mobile phase A was 100% water with 100 mM ammonium formate (pH = 3), and B was 100% acetonitrile. The column temperature was set at 45 °C and the injection volume was 2 µL. The source parameters applied operating in positive electrospray ionization (ESI+) are shown in Supplementary Table S2. The MRM transitions used, as well as the retention time for each compound, are summarized in Supplementary Table S3. The absolute value of the slope of the histamine consumption (30–120 min) represented in nmol was used to determine DAO activity in mU (nmol/min) and expressed relative to the vehicle group.

**Cell Culture and treatments**

The human colonic epithelial cell line Caco-2 (passages 35 to 40), obtained from American Type Culture Collection (ATCC), was maintained at 37 °C in a 5% CO_2_ 95% humidified air atmosphere in Dulbecco’s Modified Eagle Medium (DMEM) containing 25 mM glucose and supplemented with 15% inactivated (56 °C for 30 min) fetal bovine serum (FBS), 0.1 mM non-essential amino acids, and 50 U/mL–50 μg/mL penicillin–streptomycin. Stock cultures were seeded in 75-cm^2^ culture flasks and were passaged weekly by trypsinization (0.05% trypsin–0.053 mM EDTA). For all the experiments, cells were seeded in 96-well plates (5 × 10^3^/well). The culture medium was changed every 2 days, and cells were maintained for 14 days when enterocytes were completely differentiated [28]. This cell line is widely used as an in vitro model to study intestinal function because it undergoes enterocytic differentiation spontaneously [29,30]. It was selected for this study because of its capacity to express DAO and secrete it to the medium [31].

Based on the concentration ranges for methylphenidate and lisdexamfetamine described in the literature [32,33], it was prepared a stock solution to treat the enterocytes considering the molecular weight, the dose of the active compound and the weight of a pill. The pills were ground into a fine powder and powder drugs were solubilized with DMSO as a carrier. Then, stock solutions were filtered with a 0.22 µm membrane filter and were stored at −80 °C until they use in the following determinations.

**Cell Viability Measurement**

Viability was measured by an MTT (3-(4,5-dimethyl-thiazol-2-yl)-2,5-diphenyl-tetrazolium bromide) assay. MTT assay is a colorimetric analysis that measures the activity of different enzymes of viable cells, which transform the MTT compound to formazan, which is insoluble and has a purple color [2]. In a 96-well plate, 10,000 cells per well were cultivated in 100 µL 24 h before the treatments, allowing their adherence to the bottom of the well. Cells were rinsed twice with DMEM FBS-free culture medium prior to a 24 h incubation in the presence of three increased concentrations of the different pharmacological treatments. At the end of the 24 h incubation period, treatments were aspirated and MTT was added to each well (1 mg/mL). Cells were incubated for 2 h at 37 °C, the medium was removed, and the formazan precipitate was resuspended in 100 µL of isopropanol. The absorbance was read at 570 nm and 650 nm, as reference. The viability values of the different treatments are expressed as a relative percentage to the vehicle-treated cells. DMSO (dimethyl sulfoxide, 25%) was used as a negative control for viability. Once the working dose for each drug had been chosen, the cells were seeded in 12-well plates to carry out each of the following assays. Caco-2 cells were allowed to grow for 14 days and exposed for 24 h to the drugs of interest for the evaluation of mRNA expression, protein expression and DAO activity [34]. In addition, for each assay, an additional condition of cells treated with culture medium with the treatment vehicle will be included in the experimental design, as a negative control, and treatment with aminoguanidine, as a positive control, since it is a known DAO activity inhibitory agent [35,36]. All experimental conditions were performed at least in triplicate.

**RNA Extraction and Quantitative Polymerase Chain Reaction (RT-qPCR)**

Treated cell homogenates from Caco-2 enterocytes were used for total RNA extractions using TriPure reagent (Roche Diagnostic, Barcelona, Spain), according to the manufacturer’s instructions [30]. RNA concentration and purity were determined using a nanophotometer (Implen GmbH, München, Germany). RNA was converted to cDNA using the High-Capacity RNA-to-cDNA Kit (Applied Biosystems, Wilmington, DE, USA). The cDNAs were diluted 1:10 before incubation with a commercial LightCycler 480 Sybr green I master on a Lightcycler^®^ 480 II (Roche Diagnostics GmbH, Manheim, Germany). Aminoguanidine was shown as a natural negative regulator for *DAO*. Primers were previously described in other studies and verified with Primer-Blast software (National Center for Biotechnology Information, Bethesda, MD, USA). Sequences of oligonucleotides used in this study were: DAO F1: 5′-CGCAGACGTGATTGTCAACT-3′; DAO R1 5′-GGATGATGTACGGGGAATTG-3′ [37]; PGK1 F1: 5′-CAAGAAGTATCTGTCA-3′; PGK1 R1: 5′-CGAAGGTGGAAGAGTGGGAGTTG-3′ [38].

**Protein Extraction and Western Blot Analysis**

The selected drugs—14 days differentiated and 24 h treated—were homogenized with 100 µL lysis buffer (8 mmol/L NaH_2_PO_4_, 42 mmol/L Na_2_HPO_4_, 1% SDS, 0.1 mol/L NaCl, 0.1% NP40, 1 mmol/L NaF, 10 mmol/L sodium orthovanadate, 2 mmol/L phenylmethylsulfonyl fluoride (PMSF), and 1% protease inhibitor cocktail 1 (Millipore Sigma, Germany)). The protein extracts were quantified by the standardized BCA method (Bio-Rad Protein Assay; BioRad, CA, USA). Protein extracts (25 µg) were electrophoretically separated on 12% SDS-PAGE and electroblotted to nitrocellulose membranes (Li-cor biosciences, NE, USA) [30]. Efficient protein transfer was monitored by Ponceau-S stain. Next; membranes were blocked (5% BSA) at room temperature and probed with specific primary antibodies (diluted 1:1000) overnight at 4 °C in 1% BSA: DAO polyclonal antibody (PA5-76708, Invitrogen, Carlsbad, CA, USA) and β-Actin (Santa Cruz Biotechnology, Inc.; Dallas, TX, USA). Thereafter, infrared fluorescent secondary antibodies anti-rabbit 680, anti-rabbit 800 and anti-mouse 680 (LI-COR Biosciences, Lincoln, NE, USA; 926-32211, 926-68071 and 926-68070, respectively) were used for detection and quantified using ImageJ [39].

**Intracellular DAO Activity in Caco-2 enterocytes**

DAO activity was measured with a Diamine Oxidase Activity Assay Kit (Sigma-Aldrich; Hamburg, Germany), according to the manufacturer’s instructions [40]. Briefly, this assay provides a straightforward method to determine DAO activity of Caco-2 cell lysates, with a detection limit lower than 1 pmol/min of activity. In the assay, DAO converts the provided substrate, yielding an intermediate and hydrogen peroxide (H_2_O_2_). H_2_O_2_ is then utilized by the DAO Enzyme Mix to generate fluorescence (excitation: 535 nm/emission: 587 nm) from the DAO Probe. Finally, the DAO activity was expressed relative to the amount of protein added in the assay. Thus, an aliquot of the enterocytes extracts was quantified by BCA, as explained in the Western Blot section.

**Statistical analysis**

For each determination, an initial exploration was carried out to rule out discrepant points within the groups. For this purpose, the Grubbs statistical test was used, using the GraphPad Prism 9 software (GraphPad Software, Inc., La Jolla, CA, USA) [39]. All data are expressed as means ± standard error of the mean (SEM). To analyze differences in the different parameters, a one-way variability analysis (One Way ANOVA) was used, followed by the Dunnett post hoc test, which was performed to analyze differences of every treatment to a vehicle mean. A probability level of *p* < 0.05 was defined as statistically significant and a probability below 0.1 was defined as trending toward significance.

## 3. Results

### 3.1. DAO Activity Was Not Affected by the Presence of Methylphenidate or Lisdexamfetamine

**Figure 1:**
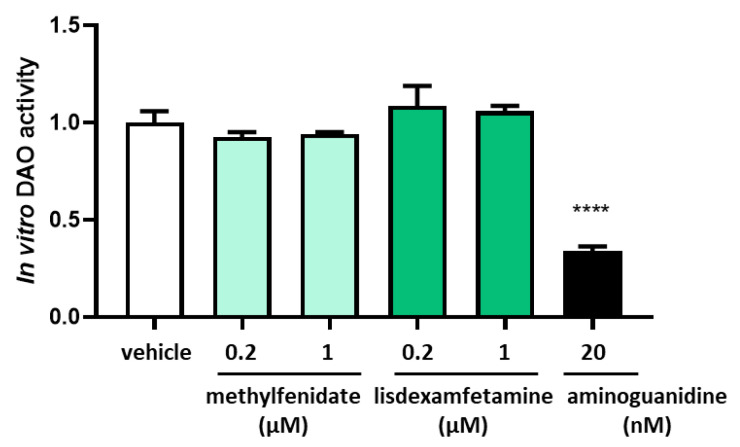
DAO activity was not affected by the presence of methylphenidate or lisdexamfetamine at the indicated concentrations. In contrast, aminoguanidine produced an important reduction of DAO activity. Data are expressed as mean ± SEM. The results are expressed relative to the vehicle group. One-way Anova test followed by Dunnett post hoc test, **** *p* < 0.0001 vs. vehicle.

LC-MS/MS chromatographic in vitro analyses showed no effects of the presence methylphenidate or lisdexamfetamine on the reduction of DAO activity, nor in low and high concentrations (Figure 1). In contrast, a strong down-regulation in the DAO activity was observed after incubation with its well-described inhibitor, aminoguanidine (Figure 1).

**Figure 2:**
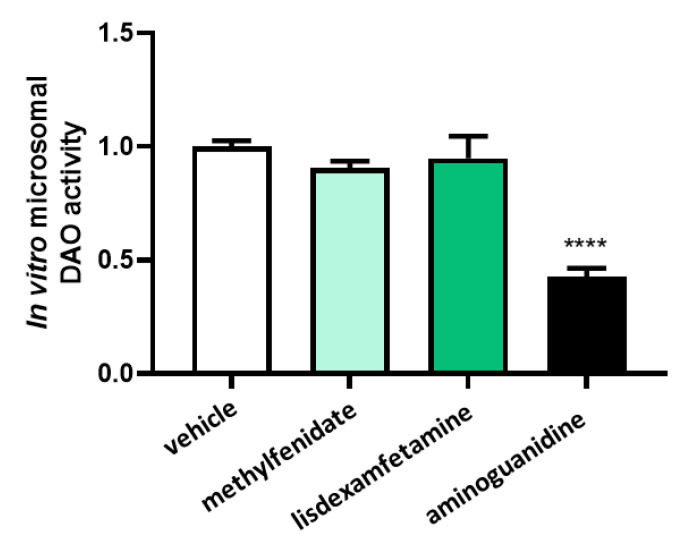
DAO activity was not affected by the presence of methylphenidate (1 µM) or lisdexamfetamine (1 µM) previously incubated with microsomes. In contrast, aminoguanidine produced an important reduction of DAO activity. Data are expressed as mean ± SEM. The results are expressed relative to the vehicle group. One-way Anova test followed by Dunnett post hoc test, **** *p* < 0.0001 vs. vehicle.

To assess whether changes in the chemical structure of methylphenidate or lisdexamfetamine after hepatic metabolization may influence DAO activity, both compounds were incubated with microsomes prior to DAO activity testing. (Figure 2). No effects in DAO activity were observed after the incubation with metabolized methylphenidate or metabolized lisdexamfetamine, whereas incubation with aminoguanidine produced an important DAO activity repression.

### 3.2. Effects of Selected Psychostimulant Drugs on Intestinal DAO

**Figure 3:**
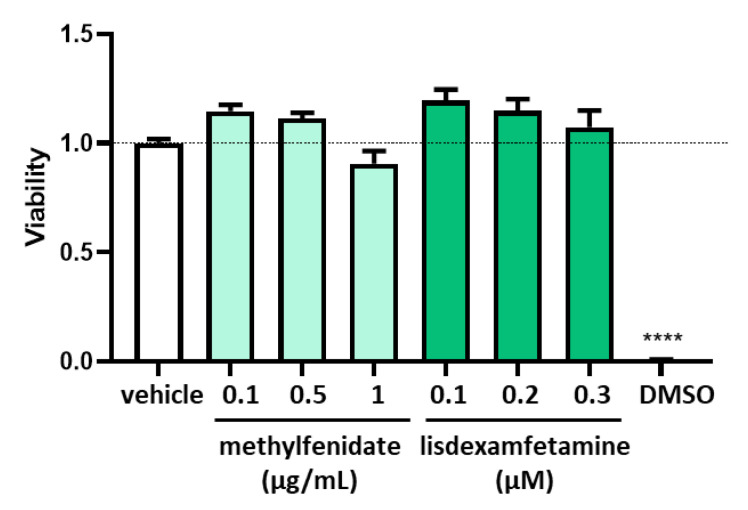
Cell viability in human enterocytes Caco-2 treated with the indicated concentrations of psychostimulant drugs (methylphenidate and lisdexamfetamine). DMSO (dimethyl sulfoxide, 25%) was used as a negative control for viability. Data are expressed as mean ± SEM (*n* = 8). The results are expressed relative to the vehicle group. One-way Anova test followed by Dunnett post hoc test, **** *p* < 0.0001 vs. vehicle.

To determine the concentrations of psychostimulant drugs in the evaluation of the potential regulation on DAO in the human enterocytes Caco-2 cell line, cell viability assays (MTT) were performed at low, medium, and high concentrations of methylphenidate and lisdexamfetamine (Figure 3). Caco-2 human enterocytes were treated with methylphenidate at concentrations of 0.1, 0.5 and 1 µg/mL, and with lisdexamfetamine at 0.1, 0.2, and 0.3 µM. No differences were observed in any of the concentrations tested of methylphenidate and lisdexamfetamine, in comparation with the vehicle treated cells. However, the higher dose methylphenidate that was tested (1 µg/mL) presented a tendency to reduce to viability of the enterocytes. Consequently, the selected methylphenidate and lisdexamfetamine concentrations to carry out the following analysis were 0.5 µg/mL and 0.3 µM for methylphenidate and lisdexamfetamine, respectively.

**Figure 4:**
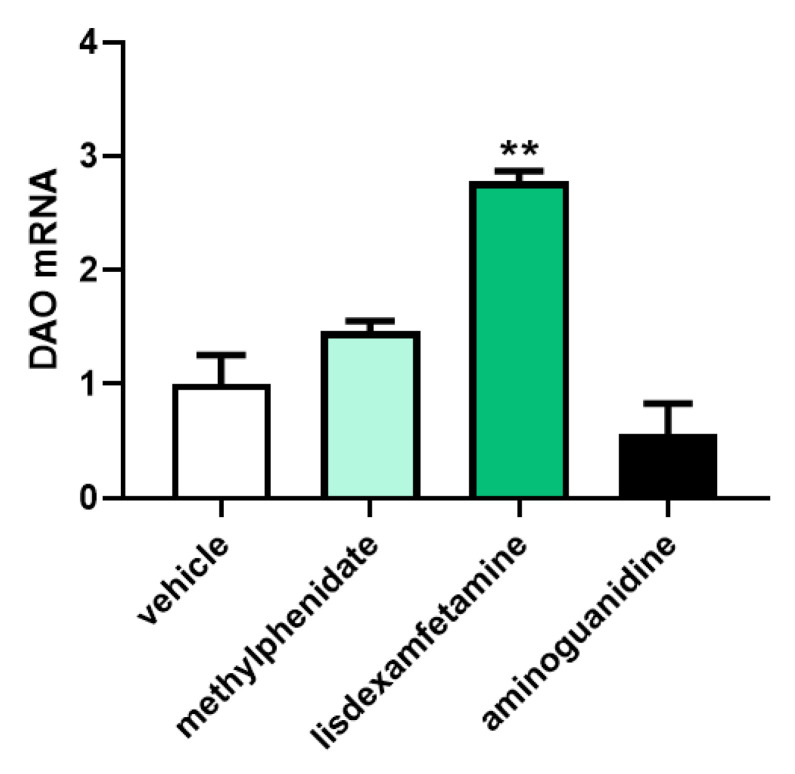
mRNA expression of DAO in human enterocytes Caco-2 treated with the psychostimulant drugs methylphenidate and lisdexamfetamine. Doses: methylphenidate (0.5 µg/mL); lisdexamfetamine (0.3 µM), aminoguanidine (10 mg/mL). Data are expressed as mean ± SEM (*n* = 3). The results are expressed relative to the vehicle group. One-way Anova test followed by Dunnett post hoc test, ** *p* < 0.01 vs. vehicle.

To determine the effect of the psychostimulant drugs, methylphenidate and lisdexamfetamine, on the gene expression of DAO in human Caco-2 enterocytes, mRNA expression assays (RT-qPCR) were performed (Figure 4). No differences were observed in the DAO mRNA expression enterocytes treated with methylphenidate or aminoguanidine. In contrast, a strong up-regulation of the DAO mRNA levels was observed after the Caco-2 enterocytes treatment with lisdexamfetamine. A similar tendency was observed with methylphenidate. These results may suggest that psychostimulant treatments could increase the DAO mRNA expression in human enterocytes, especially in the case of lisdexamfetamine.

**Figure 5:**
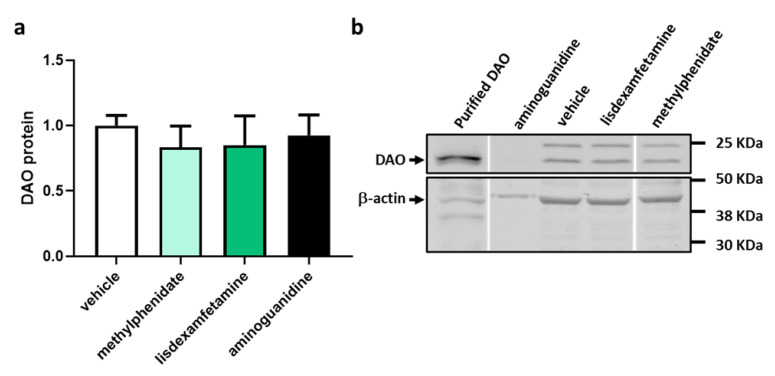
Protein expression DAO in human enterocytes Caco-2 treated with the psychostimulant drugs methylphenidate and lisdexamfetamine. (**a**) Densitometry analysis of relative DAO protein concentration after the indicated treatments. (**b**) A representative Western blot analysis of human DAO and housekeeping β-actin levels. Doses: methylphenidate (0.5 µg/mL); lisdexamfetamine (0.3 µM), aminoguanidine (10 mg/mL). The results are expressed relative to the vehicle group. Data are expressed as mean ± SEM (*n* = 3).

To determine the effect of the psychostimulant drugs, methylphenidate and lisdexamfetamine, on the protein expression of DAO in human Caco-2 enterocytes, protein expression assays (Western Blot) were performed (Figure 5). Aminoguanidine was used as a natural negative regulator for DAO activity. No effects were observed at the protein level. Protein expression of DAO did not change in enterocytes treated with methylphenidate or lisdexamfetamine. These results may suggest that psychostimulant treatments do not significantly regulate the intracellular DAO protein in human enterocytes.

**Figure 6:**
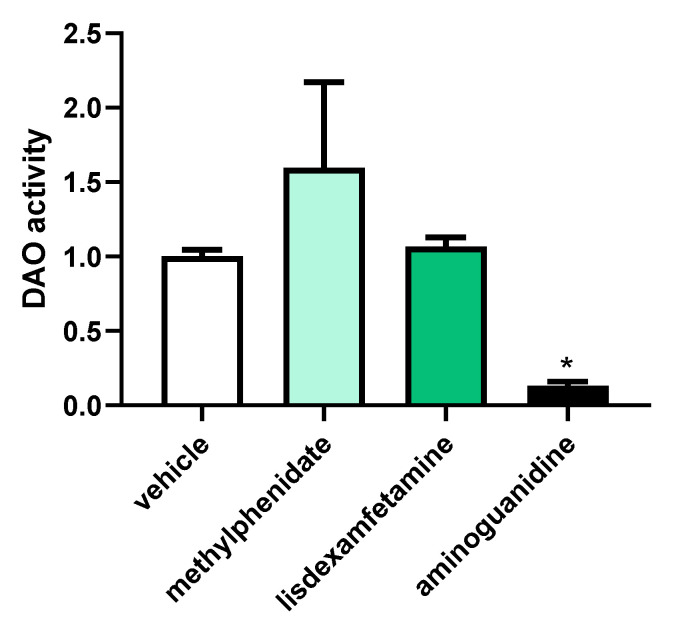
DAO activity in human enterocytes Caco-2 treated with the psychostimulant drugs methylphenidate and lisdexamfetamine. Doses: Methylphenidate (0.5 µg/mL); lisdexamfetamine (0.3 µM), aminoguanidine (10 mg/mL). Data are expressed as mean ± SEM (*n* = 3). The results are expressed relative to the vehicle group and relative to the amount of protein added in the assay. A one-way ANOVA revealed that there was a statistically significant difference in mean exam score between at least two groups (F(3, 8) = [12.77], *p* = 0.020). Dunnett’s multiple comparisons test found that the mean value of exam score was significantly different between cells treated with vehicle and cells treated with aminoguanidine (*p* = 0.017, 95% C.I. = [0.1779, 1.558]). There were no statistically significant differences between cells treated with vehicle and cells treated with methylphenidate (*p* = 0.0903) or between cells treated with vehicle and cells treated with lisdexamfetamine (*p* = 0.9857). * *p* < 0.05 vs. vehicle.

To determine the effect of the psychostimulant drugs, methylphenidate and lisdexamfetamine, on the DAO activity in human Caco-2 enterocytes, a specific DAO fluorescent activity assay was carried out (Figure 6). Although a qualitative increase in DAO activity was observed in enterocytes after incubation with methylphenidate, no significant differences were detected for either methylphenidate or lisdexamfetamine. As described in the literature [35], a strong down-regulation in the DAO activity was observed in enterocytes treated with the aminoguanidine. These results may suggest that psychostimulant treatments do not modulate DAO activity in human enterocytes.

## 4. Discussion

This study supports that the stimulant treatments for ADHD do not interfere with the activity of DAO. This was demonstrated by evaluation of DAO activity in in vitro studies, as well as in a human cell line of intestinal epithelium (i.e., colonocytes). The prevalence of ADHD is raising in different countries [41,42]. Fortunately, combination of psychostimulant drugs with behavioral change programs is an effective therapy for this disease [3]. However, there are increasing evidences describing higher histamine levels in patients suffering ADHD, which may be related with the common allergy-like symptoms presented in these patients [11]. For this reason, nutritional advice and DAO enzymes supplementation should reduce histamine levels, which would translate into a better quality of life for those ADHD patients suffering with histamine intolerance [10]. DAO supplementation could be a promising strategy in ADHD patients with DAO deficiency or HIT, compatible with current pharmacological protocols. DAO secretion follows a circadian rhythm, with higher levels of DAO activity during the day and lower levels at night [43]. This circadian rhythm is thought to be regulated by the hypothalamus, which regulates a variety of physiological processes in the body, including sleep-wake cycles. In fact, DAO supplements are more effective if taken during the day before meals, when DAO activity is naturally higher, rather than at night [44]. The reason is that the presence of DAO in the gut during the day is required when histamine-rich food is consumed, in order to degrade histamine and prevent its absorption. However, the implications of this rhythmicity for stablishing the posology of DAO supplementation, especially in ADHD patients characterized by disruption of circadian rhythm [45], provides growing evidence that disruptions of the circadian rhythm may play a role in the development and progression of ADHD [46], disturbing the sleep-wake cycle by delaying sleep onset, shortening sleep duration, and increasing daytime sleepiness. Stimulant medications used to treat ADHD, such as methylphenidate and amphetamines, have a short half-life, and are administered with delayed-release formulas once in the morning or, if necessary, twice a day doses, in the morning and midday [3,47]. Three doses per day are restricted to immediate release methylphenidate formulations in very young children for a short time, recommending to switch as soon as possible to single-dose formulas due its tendency to provoke addiction [48]. This posology should be compatible with DAO supplementation. In fact, DAO posology could be simplified in view of the observed results of the present work, with an up-regulation of DAO mRNA levels by lisdexamfetamine and a trend to increase the DAO activity by methylphenidate. Thus, administering psychostimulant medication in the morning should allow to maintain DAO at later times of the day. This regime would facilitate compliance and adherence to a chronic dietary management in a population in which it could be challenging (children), and thus compromise efficacy.

A limitation of the present study is that cultured cells grown in vitro may not accurately reflect the behavior of cells in vivo, limiting the translation of our findings to humans. This is because cells in vivo interact with other cells, the extracellular matrix, and the environment in a complex and dynamic manner that cannot be easily replicated in vitro. Additionally, cells in culture may undergo changes in gene expression or behavior due to the artificial conditions of the culture environment, such as the absence of physical forces or the presence of growth factors that are not present in vivo. Further research in preclinical models recapitulating the microenvironment found in vivo may allow researchers to overcome these limitations. In addition, results derived from this work should be validated in clinical trials targeting ADHD patients.

## 5. Conclusions

In summary, results of the psychostimulant treatments with methylphenidate and lisdexamfetamine did not present an inhibitory effect on the activity or levels of DAO, neither in vitro nor in human enterocytes. Indeed, data from mRNA expression studies and from DAO activity analysis suggest an increase in DAO levels following exposure to psychostimulant drugs. These data support clinical prescription for the concomitant use of psychostimulant medication and DAO enzyme supplementation in ADHD patients with DAO deficiency.

## Data Availability

The data presented in this study are available on request from the corresponding author.

## Supplementary Materials

The following supporting information can be downloaded at: https://www.mdpi.com/article/10.3390/jcm12144666/s1; Table S1: Gradient of the chromatographic separation; Table S2: Parameters applied in positive electrospray ionization (ESI) and Table S3: Optimized MRM transition for each analyte.
